# Fractal Nature of Metallic and Insulating Domain Configurations in a VO_2_ Thin Film Revealed by Kelvin Probe Force Microscopy

**DOI:** 10.1038/srep10417

**Published:** 2015-05-18

**Authors:** Ahrum Sohn, Teruo Kanki, Kotaro Sakai, Hidekazu Tanaka, Dong-Wook Kim

**Affiliations:** 1 Department of Physics, Ewha Womans University, Seoul 120-750, Korea; 2 The Institute of Scientific and Industrial Research, Osaka University, Osaka 567-0047, Japan

## Abstract

We investigated the surface work function (*W*_*S*_) and its spatial distribution for epitaxial VO_2_/TiO_2_ thin films using Kelvin probe force microscopy (KPFM). Nearly grain-boundary-free samples allowed observation of metallic and insulating domains with distinct *W*_*S*_ values, throughout the metal–insulator transition. The metallic fraction, estimated from *W*_*S*_ maps, describes the evolution of the resistance based on a two-dimensional percolation model. The KPFM measurements also revealed the fractal nature of the domain configuration.

Some metal oxides exhibit first-order phase transitions, in which different electronic and magnetic phases coexist during the transition^1^. Interplay among the charge, spins, lattice, and orbitals influences the spatial distribution of the different phase domains. Evolution of such domain maps under external stimuli, including heat^2^,^3^, electric and magnetic fields^2^,^3^,^4^,^5^,^6^,^7^,^8^, optical excitation^9^,^10^, and strain^11^,^12^,^13^,^14^,^15^, results in a huge change in the electrical resistance and/or optical response; one of the most notable examples is the colossal magnetoresistance in manganite thin films^2^,^8^. Theoretical studies of these fascinating phenomena have suggested that the oxides are intrinsically inhomogeneous on the nanoscale, owing to strong electronic correlations^16^.

VO_2_ exhibits metal–insulator transitions (MITs) and structural phase transitions near room temperature^1^. The coexistence of metallic and insulating domains in VO_2_ thin films and its influence on the physical properties have been investigated extensively^3^,^6^,^7^,^9^,^10^,^11^,^12^,^13^,^14^,^15^,^16^,^17^,^18^,^19^,^20^,^21^,^22^,^23^,^24^,^25^,^26^,^27^,^28^,^29^,^30^,^31^. Analyses of the macroscopic physical properties of VO_2_ often require careful consideration of the geometric configuration of the domains. Recently, Ramíez *et al.* and Aliev *et al.* reported anomalous behavior in AC impedance spectra of VO_2_ thin films^22^,^23^. These experimental data suggest that ultra-thin, highly conductive domains exist below the transition temperature, and that the domain boundaries have a fractal nature near the transition temperature. According to percolation theory, metallic clusters form a fractal surface^32^, which can explain the impedance spectra; however, there has been no explicit experimental evidence for this to date. The domain configurations significantly affect the phase transition behavior of VO_2_ thin films, as well as the performance of prototypical devices based on VO_2_ thin films, including a split-ring-resonator-based metamaterials^17^, active terahertz nanoantennas^18^, ultra-thin perfect absorbers^19^, and phase-transition-driven memristive systems^20^.

Thermal and lattice mismatch between films and substrates can induce tensile or compressive strain, and accumulated elastic distortion often generates grain boundaries (GBs) in thin films. It has been reported that the phase transition behaviors of VO_2_ thin films largely vary depending on their strain states^12^,^15^. Moreover, GBs in VO_2_ thin films show distinct conduction properties compared with the intra-grain region^25^. Recently, several research groups successfully prepared very thin, nearly GB-free VO_2_ thin films on TiO_2_ substrates, taking advantage of the small lattice mismatch^7^. In this regard, VO_2_/TiO_2_ thin films provide an ideal system for studying the intrinsic two-dimensional (2D) phase transition characteristics of VO_2_.

All of these considerations raise the following question: How can we directly observe the metallic and insulating domain configurations of VO_2_ thin films and their evolution throughout the transition? Conventional x-ray diffraction (XRD), photoemission spectroscopy, and optical spectroscopies can be used to investigate the physical properties of VO_2_ during the MIT^21^; however, these techniques provide only the physical quantities averaged at the macroscopic scale. Nanoscale probe-based tools, such as scanning tunneling microscopy (STM) and scanning probe microscopy (SPM), have superior spatial resolution. Various SPM-based techniques have been developed to probe the electrical properties of samples. In particular, Kelvin probe force microscopy (KPFM) is a versatile tool for investigating the local surface potential under ambient conditions^31^,^33^.

In this work, we demonstrated that KPFM measurements of the local surface work function (*W*_*S*_) can be used to reveal the intriguing phase transition behaviors of the VO_2_/TiO_2_ thin films. The *W*_*S*_ maps clearly show the spatial distribution of metallic and insulating domains during the transition. The evolution of the metallic domain fraction well explains the temperature dependence of the resistivity based on the 2D percolation model. The domain size is tens of nanometers at intermediate temperatures. The perimeter and area of the metallic domains follow power-law behaviors: a power exponent larger than 1/2 suggests that the metallic domains are fractal objects.

Figure 1 shows the XRD pattern for the 15-nm-thick VO_2_ thin film grown on a TiO_2_ (001); two significant peaks corresponding to tetragonal VO_2_ (002) at the higher angle and TiO_2_ (002) at the lower angle were evident. The cross-sectional high-resolution transmission electron microscopy (HR-TEM) image shown in the inset of Fig. 1 confirms the epitaxial crystallinity. Previously, we reported details of the sample preparation procedures and characterization results^7^.

Figure 2a shows a schematic diagram of the measurement setup. Transport and KPFM measurements (XE-100, Park Systems) can be simultaneously performed in a glove box filled with N_2_ gas. The sample temperature was varied from 285 to 355 K using a sample stage equipped with a Peltier device. The VO_2_ thin film was patterned by photolithography and reactive ion etching with SF_6_ gas. The width and length of the stripe-shaped pattern were 4 and 30 μm, respectively.

Figure 2b shows the resistivity (*ρ*) and surface work function (*W*_*S*_) measured during a heating and cooling cycle. *W*_*S*_ was averaged over a 2 × 1-μm^2^ scanned area on the VO_2_ surface. *ρ* undergoes an abrupt change around 300 K. The transition temperature (*T*_*C*_) is less than that of a single crystal, due to the shorter *c*-axis length caused by epitaxial strain^28^. *W*_*S*_ gradually decreases (increases) while raising (lowering) the sample temperature, indicating variation in the electronic structure. The temperature dependences of both *ρ* and *W*_*S*_ were reproducible under repeated heating and cooling cycles. Thus, although surface redox must be accounted for under ultra-high-vacuum conditions^29^, it was not a consideration in this study.

Figure 3a-d show the *W*_*S*_ maps obtained from an identical region, whose morphology is shown in Fig. 3e, while heating the sample from 285 to 355 K. The sample surface was very flat; the root-mean-square roughness was only 0.42 nm. Hence, topographic artifacts had little effect on the *W*_*S*_ images. From Fig. 2b, two values (i.e., 5.06 and 4.96 eV) can be chosen as representative values for low- and high-temperature *W*_*S*_, respectively. The two values correspond to blue and red colors in the *W*_*S*_ maps shown in Fig. 3a–d; as the temperature increased, the area of the red (blue) region decreased (increased). Note that the VO_2_ surface regions were either red or blue in color only, with the exception of the boundary region between the two colors. In contrast, our earlier study showed that the VO_2_/Al_2_O_3_ films exhibited a gradual change of *W*_*S*_ over the entire surface area with variations in the sample temperature^31^. The VO_2_/Al_2_O_3_ films suffer from rather large strain due to the lattice mismatch, generating high-density GBs and tens-of-nm-sized grains. The domain size should be limited by the GBs and the width of the space charge region (SCR) formed at the boundaries of the metallic and insulating domains, which can cover large portions of the sample surface area during the transition^31^. Consequently, we expect that band bending at the domain boundaries would dominate the spatial distribution and temperature evolution of *W*_*S*_ in the VO_2_/Al_2_O_3_ film with small grains. A comparison of VO_2_/TiO_2_ (this work) and VO_2_/Al_2_O_3_ (Ref. 31) thin films showed that distinct strain states and resulting microstructures can significantly influence the evolution of the *W*_*S*_ maps in the VO_2_ thin films.

Kelvin probe force microscopy is based on the electrostatic interaction between the probe tip and sample, and has a spatial resolution of tens of nm^34^. Some of the intermediate regions shown in Fig. 3a–d might be caused by the limited spatial resolution of KPFM; however, the dimensions of some of the intermediate-work-function regions (shown by the green regions in Fig. 3a–d) are significantly larger than the spatial resolution limit and red spots can be seen at the center of such green regions. The limited resolution alone may not explain such results. Therefore, the intermediate regions in the work function maps should be explained by the SCR formation at the domain boundaries as well as the resolution limit of KPFM.

Near-field scanning optical microscopy (NSOM)^3^ and hard x-ray nanoprobe (HXN) measurements^30^ revealed metallic and insulating domains in the VO_2_ thin films as they underwent the MIT. In both NSOM and HXN data, the spectra and diffraction patterns near *T*_*C*_ were broader than those far from *T*_*C*_, implying the coexistence of the two phases; however, extraction of a physical parameter from individual domains is not straightforward. The dielectric constant from optical spectra and the lattice constant from diffraction patterns can be obtained only after model-based fittings, in which the associated sample states and fitting parameters are chosen subjectively. In contrast, KPFM results provide the local surface work function of a specific area without the need for numerical analysis.

From the map for each temperature, the metallic region can be identified as the region with the high-temperature *W*_*S*_ (*i.e.*, the blue-colored region in Fig. 3a–d). The typical size (tens of nm) and the shape of the metallic domain were similar to those obtained from the NSOM and HXN results^3^,^30^. The area with the high-temperature *W*_*S*_ enables us to estimate the metallic fraction, *P*_*M*_. *P*_*M*_ increases linearly with the sample temperature. Interestingly, the metallic domains (the blue region) were present even at 285 K, far below *T*_*C*_ (Fig. 3a). Ramirez *et al.* suggested the existence of persistent metallic domains in VO_2_ from analysis of the resistance hysteresis during the MIT^27^. Additionally, a small part of the sample surface had a low-temperature *W*_*S*_ even at 355 K, far above *T*_*C*_(the red region in Fig. 3d); this may correspond to an area where stain relaxation occurs, stabilizing the insulating phase at high temperatures^26^.

Figure 4a and 4b show the difference in *W*_*S*_ between high and low temperatures. The average *W*_*S*_ decreased as the temperature increased (see Fig. 2b), and hence we would expect local *W*_S_ to be smaller at high temperatures than at low temperatures (see the dark color in Fig. 4a and 4b). From Fig. 4a and 4b, however, in some localized regions (see light colored regions) we can see that *W*_S_ increased with temperature. This suggests that an inverse phase transition (i.e., from insulating to metallic phases) occurred in these local areas. Qazilbash *et al.* observed a similar inverse phase transition at the nanoscale in HXN experiments^30^. Our data appear to show relevant experimental results, although a clear understanding of the physical origin is currently lacking.

Figure 5a shows the full-width-half-maximum (FWHM) of the *W*_*S*_ distributions as a function of temperature. Figure 5b shows histograms of *W*_*S*_ at 285, 305, and 355 K; *P*_*M*_ gradually increased with the sample temperature, as expected from the progressive decrease in *W*_*S*_ (see Fig. 1c). The FWHM exhibited a broad single peak centered at 305 K, corresponding to a *P*_*M*_ value of nearly 1/2. When the metallic and insulating domains have nearly the same area, *W*_*S*_ will experience its highest standard deviation, and the FWHM was a maximum.

Figure 6 shows the sample conductivity, *σ*, as a function of *P*_*M*_ obtained from the *W*_*S*_ maps. *σ*, increases abruptly at *P*_*M*_ ~ 0.3. The percolation model can be applied to describe the relationship between *σ*, and *P*_*M*_, as reported previouly^7^,^21^. According to the percolation model, *σ*, should have a power-law dependence as a function of *P*_*M*_, *i.e.*, *σ*, ∝ (*P*_*M*_ – *P*_*C*_)^*t*^, where *P*_*C*_ is the percolation threshold and *t* is the critical exponent^32^. The universal values are *P*_*C*_ = 0.45 and *t* = 1.4, for the 2D percolation conduction model, and 0.15 < *P*_*C*_ < 0.17 and *t* = 2.0, for the 3D percolation model^7^,^21^,^32^. The *P*_*M*_ dependence of *σ*, can is described well by the 2D percolation model with *P*_*C*_ = 0.36 and *t* = 1.46, as shown in the inset of Fig. 6. These values are similar to those reported from optical microscopy measurements^7^ of epitaxial VO_2_/TiO_2_ thin films. These results suggest that the value of *P*_*M*_ from our KPFM measurements accurately describes the metallic fraction in the VO_2_/TiO_2_ thin films.

Chang *et al.* reported that *P*_*M*_, estimated from scanning tunneling spectroscopy (STS) experiments deviated significantly from that obtained from XRD data^21^. STS is a technique used to study the surface density of states; atomic resolution can be achieved with this technique for well-prepared surfaces. VO_2_ has very strong electron-lattice coupling and correlation effects; hence, the surface physical properties can be altered significantly from the bulk properties^21^. However, the surface preparation procedures to obtain atomically ordered oxide surfaces have not been well established^35^. In particular, it can be difficult to achieve oxygen stoichiometry at the oxide surface in ultrahigh vacuum^29^, thus, inherent features of metal oxides can limit observation of intrinsic MIT behavior in VO_2_ using STS analysis.

Figure 7 shows that the relationship between the perimeter (*Σ*) and area (*A*) of metallic domains at 285, 305, and 335 K follows a power law; specifically, log(*Σ*) ∝ log(*A*). More interestingly, the data for the three temperatures overlapped a single line. The power exponent, *i.e.*, the slope in the log(*Σ*)-log(*A*) plot, was estimated to be 0.74. Objects with Euclidian shapes should have a slope of 0.5, because *A* ∝ *Σ*^2^. Thus, the larger exponent suggests that the metallic domains are fractal objects, having a fractal dimension, *D* = 1.48 (i.e., *Σ* ∝ *A*^1.48/2^). The percolation cluster is known to be an example of a random fractal^32^, and our KPFM measurements provide direct evidence for the fractal nature of the domain shapes in VO_2_.

Simultaneous measurements of *W*_*S*_ and the resistivity of VO_2_/TiO_2_ thin films in this study provided information on the temperature-dependent domain configurations and their influence on MIT behaviors. The local surface *W*_*S*_ of a specific area was measured directly using KPFM, without the need for additional numerical analysis. *W*_*S*_ maps, obtained by KPFM, showed that the nearly GB-free VO_2_/TiO_2_ thin films had tens-of-nm-sized metallic and insulating domains with clearly distinct *W*_*S*_ values, throughout the MIT transition. The 2D percolation model well explains the relationship between the metallic domain fraction and the sample resistivity. Real-space domain maps also suggest that the domains form a fractal surface, which is a well-known feature of percolation clusters.

## Methods

VO_2_ thin films that were 15-nm-thick were deposited on rutile TiO_2_ (001) substrates using pulsed laser deposition using an ArF Excimer laser with a wavelength of λ = 193 nm, a repetition rate of 2 Hz, and a fluence of 10 mJ cm^**−**2^ at 430** **°C in an 1.0-Pa oxygen atmosphere. A V_2_O_5_ pellet was used as the target, and the deposition rate was approximately 0.3 nm min^**−**1^. KPFM measurements were made using an atomic force microscope (XE-100, Park Systems) with a glove box. The glove box was purged using N_2_ for more than 3 hours, and the sample was then heated to 100 °C for 30 min to remove any adsorbed water. The KPFM and transport measurements were then carried out while varying the sample temperature using a Peltier device. Conductive Pt-coated Si cantilevers with a resonance frequency of ~240 kHz (NSG10/Pt, NT-MDT) were used to characterize the work function and topography. Immediately following each measurement, the work function of the tip was calibrated using highly ordered pyrolytic graphite (HOPG) (SPI Supplies) as a reference sample. The measurements were repeated several times and no noticeable differences were observed, confirming the reproducibility of the data. For the transport experiments, Al wires were bonded at the ends of a striped pattern using a wire bonder (7476D, West Bond). Two-probe electrical measurements were performed using a semiconductor parameter analyzer (4156B, Hewlett Packard) simultaneously with the work function measurements.

## Author Contributions

A.S. and D.K. performed the measurements and analyzed the data. T.K., K.S. and H.T. prepared and characterized the samples. All authors discussed the results and commented on the manuscript.

## Additional Information

**How to cite this article**: Sohn, A. *et al*. Fractal Nature of Metallic and Insulating Domain Configurations in a VO_2_ Thin Film Revealed by Kelvin Probe Force Microscopy. *Sci. Rep.* **5**, 10417; doi: 10.1038/srep10417 (2015).

## Figures and Tables

**Figure 1:**
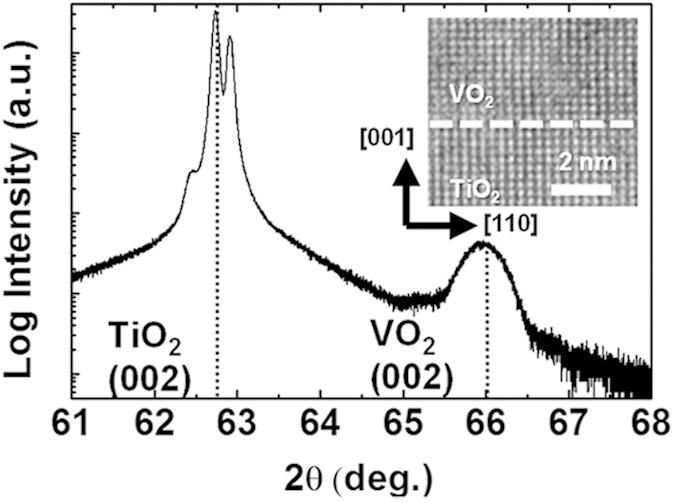
An x-ray diffraction (XRD) pattern of a 15-nm-thick VO_2_ thin film grown on a TiO_2_(001) substrate. The inset shows a cross-sectional high-resolution transmission electron microscopy (HR-TEM) image of the VO_2_/TiO_2_ thin film.

**Figure 2:**
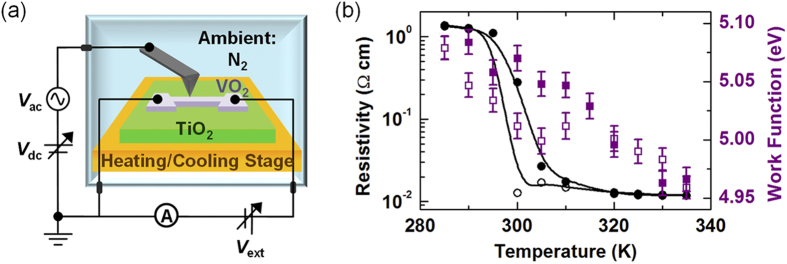
(**a**) Schematic illustration of the transport and Kelvin probe force microscopy (KPFM) measurement setup. (**b**) The resistivity (circles) and work function (squares) as a function of temperature for the VO_2_/TiO_2_ film. The filled symbols indicate data obtained during heating cycles, and the open symbols indicate data obtained during cooling cycles.

**Figure 3:**
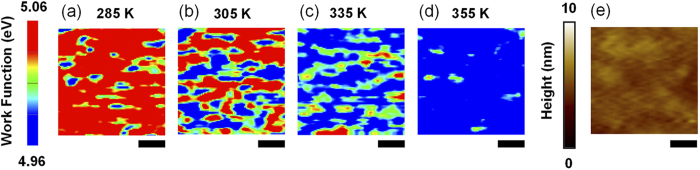
Work function maps at (**a**) 285, (**b**) 305, (**c**) 335, and (**d**) 355 K during heating. The color scale is the same for all images. The larger work function (red) represents the insulating phase and the smaller work function (blue) represents the metallic phase. (**e**) The surface morphology of the region used to obtain the work function maps. The scale bar is 100 nm.

**Figure 4:**
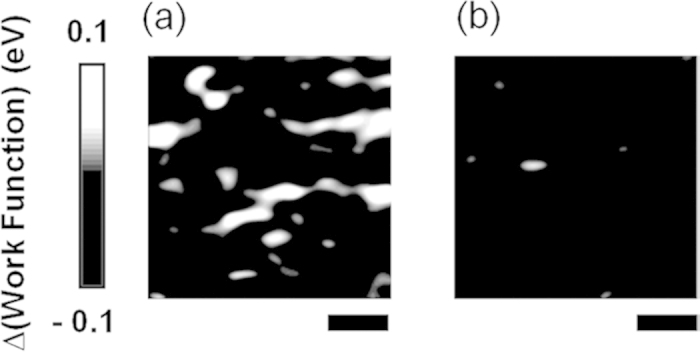
Maps of the difference in the work function (*W*_*S*_) at different temperatures. (**a**) *W*_*S*_(305 K)—*W*_*S*_(285 K) and (**b**) *W*_*S*_(355 K)—*W*_*S*_(285 K). The scale bar is 100 nm.

**Figure 5:**
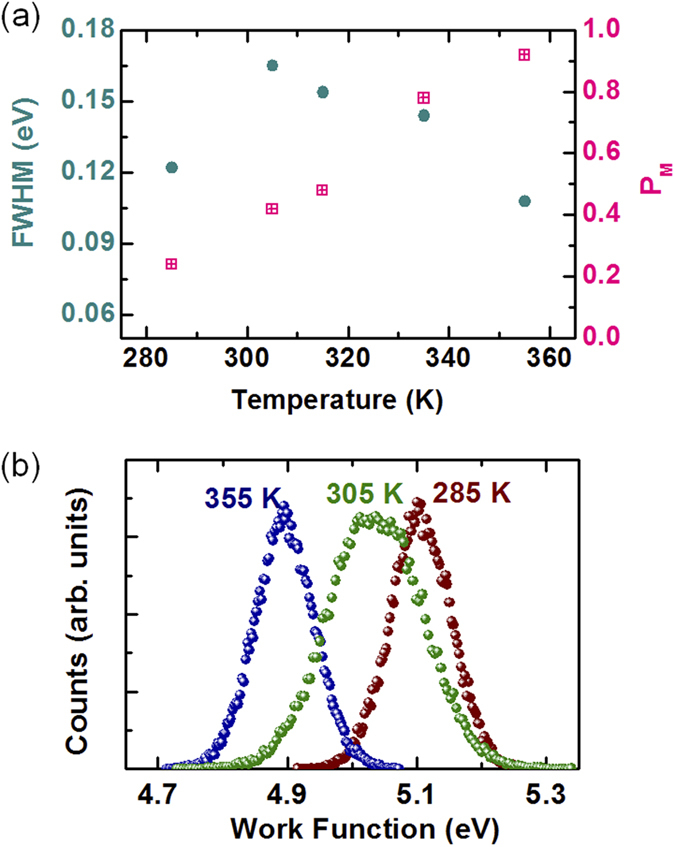
(**a**) The full-width-half-maximum (FWHM) of the work function distributions and metallic fraction (*P*_*M*_) as a function of temperature. (**b**) Work function histograms for sample temperatures of 285, 305, and 355 K.

**Figure 6:**
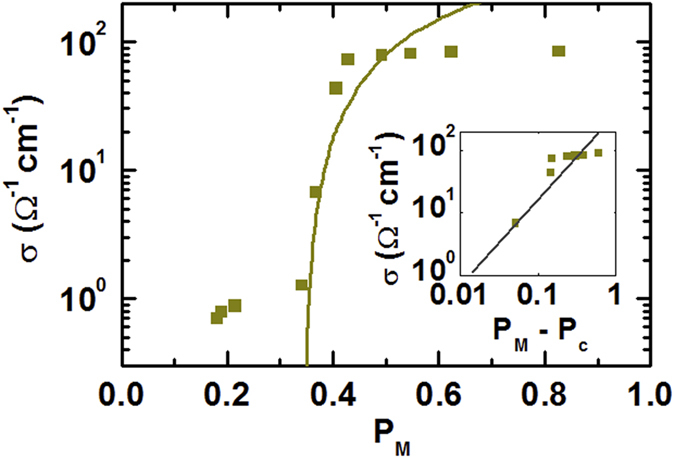
The conductivity, *σ*, as a function of *P*_*M*_ during heating. The solid line fits the data correspond to the two-dimensional (2D) percolation model, *σ*, ∝ (*P*_*M*_ – *P*_*C*_)^*t*^; the inset shows a log-log plot of *σ*, versus (*P*_*M*_ – *P*_*C*_), where *P*_*C*_ is the percolation threshold.

**Figure 7:**
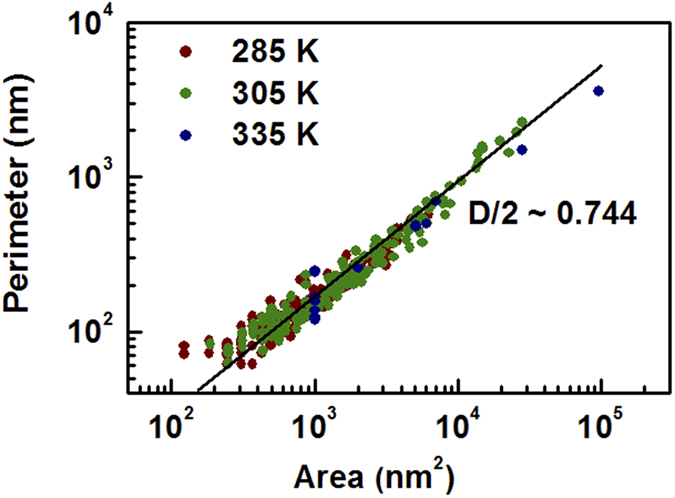
Log-log plots of the perimeters (*Σ*) of the metallic domains as a function of their area (*A*) at 285 K (<*T*_*C*_), 305 K (~*T*_*C*_), and 335 K (>*T*_*C*_). A single fit well describes the power-law behavior between *Σ* and *A* obtained at these temperatures. The slope of the log(*Σ*)-log(*A*) plot was 0.74; a value greater than 0.5 indicates a fractal nature of the domain shapes.
